# MULTISTAKEHOLDER COLLABORATION TO BETTER ADDRESS MEANING IN LIFE: PARTICIPATORY ACTION RESEARCH

**DOI:** 10.1093/geroni/igac059.1425

**Published:** 2022-12-20

**Authors:** Franka Bakker, Theo van Leeuwen

**Affiliations:** Windesheim University of Applied Sciences, Zwolle, Overijssel, Netherlands; Department of Theology and Philosophies of Life, Windesheim University of Applied Sciences, Zwolle, Overijssel, Netherlands

## Abstract

Meaning in life of older adults is related to a good life. Health and social care professionals are expected to address tensions in finding meaning in life among their clients. From 2020 to 2022, we conducted a 24-month participatory action research to discover how professionals in spiritual care, social workers, and volunteers can collaborate to better recognize, address and support questions regarding meaning in life. Four groups of co-researchers were composed, each consisting of a social worker, professionals in spiritual care, older adult, volunteer, students and researcher. They had monthly meetings to discuss actions and findings. In-between, collaboration activities were shaped and qualitative data were gathered about e.g. experiences. Results show that collaboration can be shaped by activities like providing workshops, expert consultation, coaching-on-the-job, jointly shaped interventions for clients, dialogue sessions, and multidisciplinary meetings. Thereby, for realising sustainable collaboration and ongoing mutual learning, structural and person-independent encounters should be realised.

